# Gel-Based Self-Powered Nanogenerators: Materials, Mechanisms, and Emerging Opportunities

**DOI:** 10.3390/gels11060451

**Published:** 2025-06-12

**Authors:** Aditya Narayan Singh, Kyung-Wan Nam

**Affiliations:** 1 Department of Energy and Materials Engineering, Dongguk University—Seoul, Seoul 04620, Republic of Korea; aditya@dongguk.edu; 2 Center for Next Generation Energy and Electronic Materials, Dongguk University—Seoul, Seoul 04620, Republic of Korea

**Keywords:** self-powered nanogenerators, piezoelectric, wearable electronics, energy harvesting, flexible sensors

## Abstract

With the rapid rise in Internet of Things (IoT) and artificial intelligence (AI) technologies, there is an increasing need for portable, wearable, and self-powered flexible sensing devices. In such scenarios, self-powered nanogenerators have emerged as promising energy harvesters capable of converting ambient mechanical stimuli into electrical energy, enabling the development of autonomous flexible sensors and sustainable systems. This review highlights recent advances in nanogenerator technologies—particularly those based on piezoelectric and triboelectric effects—with a focus on soft, flexible, and gel-based polymer materials. Key mechanisms of energy conversion are discussed alongside strategies to enhance performance through material innovation, structural design, and device integration. Special attention is given to the role of gel-type composites, which offer unique advantages such as mechanical tunability, self-healing ability, and biocompatibility, making them highly suitable for next-generation wearable, biomedical, and environmental sensing applications. We also explore the evolving landscape of energy applications, from microscale sensors to large-area systems, and identify critical challenges and opportunities for future research. By synthesizing progress across materials, mechanisms, and application domains, this review aims to guide the rational design of high-performance, sustainable nanogenerators for the next era of energy technologies.

## 1. Introduction

Imagine your morning coffee starts brewing as you wake up, a notification pops up for medication, and your thermostat regulates a pleasing temperature automatically to the changing weather—all seemingly orchestrated by the Internet of Things (IoT). With the global proliferation in IoT devices, there is a growing demand for flexible sensing technologies, including implantable medical devices, electronic skin, and wearable electronics [1,2,3,4,5]. To realize the practical deployment of these devices, there is an urgent need to manufacture high-performance flexible sensors that are not only portable, wearable, and mechanically compliant but also self-powered. However, the rapid evolution of such modern devices cannot be sustained by conventional power technologies, such as capacitive or piezoresistive sensors, which rely on external power sources, or even by certain piezoelectric sensors that are limited by material constraints [6,7,8].

Triboelectric nanogenerators (TENGs) are an ideal choice for the development of such sensors and are based on contact-type electrostatic induction and electrification [9,10]. TENGs are gaining significant attention from the research community owing to their benefits that include, but are not limited to, being of compact size, economical, available in wide range of materials, having high sensitivity, and being self–powered [11,12]. Apart from this, materials engaged in flexible sensors must meet certain material constraints, such as that they must be biocompatible, flexible, and environmentally benign [13]. Therefore, it is essential to explore flexible materials with desired performances to meet the requirements of TENGs for flexible sensing applications.

At the heart of self-powered nanogenerators lie two key mechanisms: piezoelectricity and triboelectricity [14]. Piezoelectricity arises from the intrinsic ability of certain materials to generate an electric charge in response to applied mechanical stress due to asymmetric displacement of ions within the crystal lattice. On the other hand, triboelectricity is a surface phenomenon that involves electron transfer between materials with differing electron affinities during repeated contact and separation. These effects are significantly pronounced at the nanoscale, where the dramatically increased surface-to-volume ratio and enhanced molecular-level interactions facilitate more efficient charge separation and transfer, thereby greatly boosting charge generation and energy conversion efficiency.

It is widely recognized that the output performance of TENGs depends quadratically on surface charge density, which can be substantially boosted by strategic material choice and advanced nanoscale engineering techniques [15]. A compelling example of this is demonstrated by Kim’s group [16], where nanoscale modification led to improved mechanical-to-electrical conversion, resulting in a significantly higher open-circuit voltage. Specifically, they employed surface-functionalized indium tin oxide (ITO) nanohelix structures arranged in an interlocked array, which provided a dramatically increased surface area and enhanced frictional contact. This innovative architecture not only amplified charge generation efficiency but also imparted excellent mechanical flexibility and durability under strain. Their TENG devices achieved over a 340-fold enhancement in output power compared to planar ITO-based systems, clearly illustrating the transformative potential of nanoscale surface engineering in advancing high-performance, flexible, self-powered electronics. This nanoscale sensitivity is what makes nanogenerators particularly suitable for flexible and miniaturized energy-harvesting systems, especially in applications such as wearable electronics, human–machine interfaces, and biomedical sensors.

In this context, soft and stretchable materials have emerged as promising candidates to address the mechanical and biocompatibility constraints associated with traditional sensor materials. Recent advances have shown that gel-type polymer composites—including hydrogels, ionogels, and organogels—are highly promising for triboelectric nanogenerators (TENGs) owing to their unique combination of softness, stretchability, and biocompatibility [17]. These gels consist of crosslinked three-dimensional polymer networks swollen with aqueous or ionic liquids, synthesized via diverse methods such as physical/chemical crosslinking, solvent exchange, and embedding conductive fillers like carbon nanotubes, graphene, and MXenes [18,19,20,21,22,23,24]. The gel’s soft and hydrated matrix allows for exceptional mechanical compliance and self-healing properties, enabling TENG devices to maintain high energy conversion efficiency under dynamic deformations including bending, stretching, and folding—capabilities often unattainable with conventional rigid polymers like PTFE or silicone elastomers. Quantitative studies [2,25] have demonstrated that gel-based TENGs achieve superior triboelectric charge densities and mechanical durability, outperforming many non-gel materials in wearable and implantable applications. Their ability to conform to irregular surfaces and sustain stable electrical output during mechanical stress underscores their potential as multifunctional platforms for next-generation flexible and self-powered sensors. As a result, gel-based materials represent a new class of multifunctional platforms that can simultaneously fulfill the mechanical, electrical, and environmental requirements of advanced self-powered flexible sensors.

Since Xu et al. [26] first used hydrogel materials for TENGs, gel-based materials have been extensively explored and studied. Despite extensive reports presenting remarkable strides of developments, the majority of these studies focused on a single type of gel, predominantly hydrogels [27]. This strong research preference can be attributed to the intrinsic advantages of hydrogels, including their high water content, tunable mechanical properties, excellent stretchability, and inherent biocompatibility [28]. These characteristics make hydrogels especially suitable for wearable, biomedical, and skin-interfacing applications, where conformability and safety are critical. Therefore, it becomes imperative to comprehensively review gel-based nanogenerators, with particular emphasis on key factors such as energy conversion efficiency, triboelectric performance, and sustainable power generation. Furthermore, their relevance to emerging technologies, including the IoT, warrants dedicated discussion. This review comprehensively summarizes recent progress in gel-based materials for nanogenerators for sensing applications from the perspective of piezoelectric and triboelectric effects—with a focus on soft, flexible, and gel-based polymer materials. Key mechanisms of energy conversion are discussed alongside strategies to enhance performance through material innovation, structural design, and device integration.

**Figure 1:**
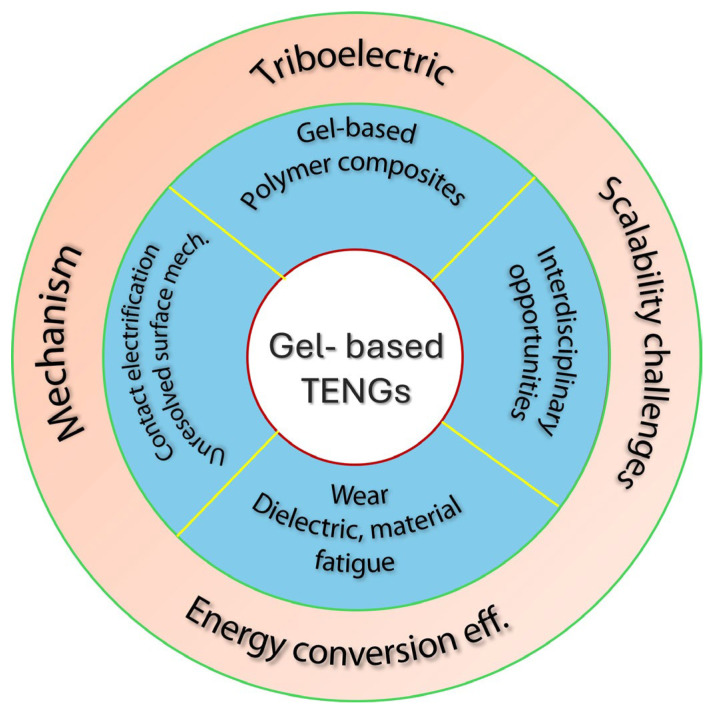
Overview of the content discussed in this review.

In this review, we briefly introduce the basic mechanisms of self-powered nanogenerators as well as basic factors that are essential to quantify the performances. Then, based on the recent progress in the field, we systematically explore the piezoelectric, triboelectric, and hybrid mechanisms, material choices, structural designs, and performance optimization strategies, which are depicted in Figure 1. Subsequently, their applications in sensing, human–machine interaction (HMI), environmental monitoring, and other fields are outlined as well. Penultimately, based on recent progress, current challenges and strategies to overcome them are proposed. We believe this review will inspire new researchers to tackle these critical challenges and explore new frontiers in their research careers.

## 2. Working Principle

### 2.1. Overview

**Figure 2:**
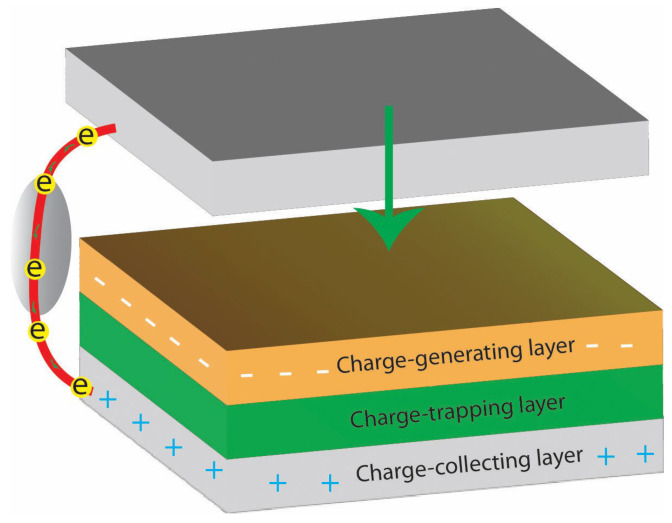
Representative schematic of a TENG component.

The working principle of nanogenerators, specifically triboelectric nanogenerators (TENGs), is based on converting mechanical energy into electrical energy through the triboelectric effect [29]. In TENGs, two materials are brought into contact and then separated, resulting in the transfer of charge between the materials due to differences in their electron affinity (Figure 2). This contact and separation generate a potential difference, which is harnessed as electrical energy.

Figure 2 illustrates the fundamental structure and key functional layers of a TENG device. At its core are two critical components: the charge-generating layer and the charge-trapping layer [29]. The charge-generating layer corresponds to the surface of the triboelectric material where electron or ion transfer occurs during contact with another material. Factors such as surface roughness, chemical composition, and surface defects influence the efficiency of this charge generation, which forms the initial separated charges essential for electrical output. Adjacent to this, the charge-trapping layer typically consists of a dielectric or insulating material that stabilizes the generated charges by capturing and holding them. This charge retention prevents immediate recombination or redistribution, maintaining a stable electrostatic potential difference that drives current flow through the external circuit. Efficient charge trapping is vital for sustaining energy conversion and improving the device’s output performance.

For gel-based TENGs, this mechanism is similar but benefits significantly from the unique properties of gel materials. The polymer networks and ionic components within gels can enhance charge transfer and retention by facilitating ion migration and improving contact intimacy under deformation states such as stretching, bending, and folding. This results in enhanced flexibility, mechanical compliance, and consistent performance in wearable and flexible electronic applications [30,31].

### 2.2. Working Modes

**Figure 3:**
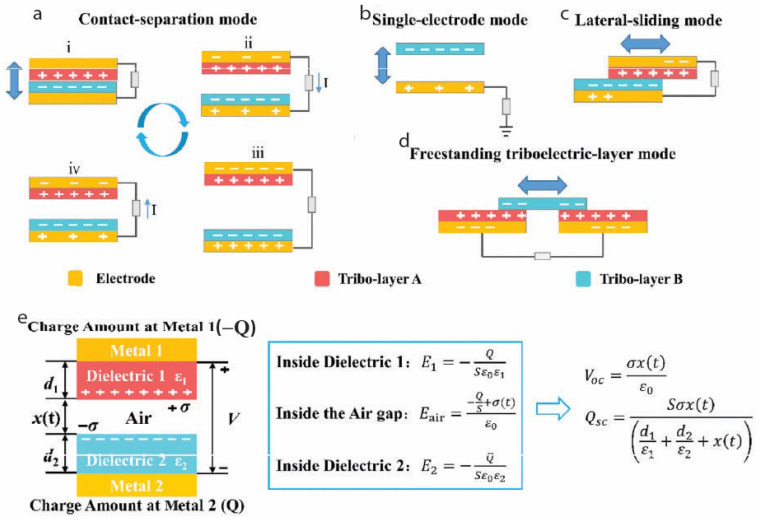
Modes of TENGs. (**a**) Vertical contact–separation (CS) mode. (**b**) Single-electrode (SE) mode. (**c**) Lateral-sliding (LS) mode. (**d**) Freestanding triboelectric layer (FT). (**e**) Schematic representation of the working of the vertical CS mode. Adapted with permission from ref. [35]. Copyright 2024, RSC.

There are four different modes of TENG operations: vertical contact-separation (CS), lateral-sliding (LS), single-electrode (SE), and freestanding triboelectric layer (FT). In the vertical contact-separation (CS) mode (Figure 3a), two different triboelectric materials repeatedly contact and separate vertically, generating alternating electric currents via induced electrostatic charges. In the lateral-sliding (LS) mode (Figure 3b), two triboelectric layers slide horizontally relative to each other, causing a periodic change in the contact area, thus inducing a charge flow and generating current pulses in an external circuit. The single-electrode (SE) mode (Figure 3c) involves only one electrode attached to a triboelectric material, relying on electrostatic induction with an external charged object or surface moving relative to it, making it particularly suitable for self-powered sensing applications where grounding may be impractical. Finally, the freestanding triboelectric layer (FT) mode (Figure 3d) features an independent charged triboelectric layer that moves freely between two stationary electrodes; this free movement of the charged layer modulates electric potential across the electrodes, effectively converting mechanical motion into electrical energy. Each mode provides unique advantages and application potentials, collectively making TENGs versatile energy-harvesting devices suitable for diverse mechanical energy sources.

To predict the o/p from these different TENGs, several theoretical models have been predicted [32,33]. Without bias, the CS model is widely regarded as the most representative (Figure 3e). In this mode, the TENG consists of two electrodes and two tribo-layers, each with different thicknesses (d_1_ and d_2_) and dielectric constants (ε_1_ and ε_2_). The distance between the layers, denoted as x(t), changes with the external mechanical force. Upon contact, equal positive and negative charges are generated on the inner surfaces of the tribo-layers. As the layers separate, an air gap forms, inducing a potential (V) between the electrodes. The transferred charge (Q) between the electrodes can be calculated using Gauss’s theorem [34]. As per Gauss’s theorem, the voltage difference across both the electrodes with the different d_1_ and d_2_ and air gap is as shown in Equation (1):

(1) $$V\left(t\right)=E_{1}d_{1}+E_{2}d_{2}+E_{\mathrm{air}}x$$

(2) $$=-\frac{Q}{S\varepsilon_{0}}\left[\frac{d_{1}}{\varepsilon_{1}}+\frac{d_{2}}{\varepsilon_{2}}+x\left(t\right)\right]+\frac{\sigma }{\varepsilon_{0}}x\left(t\right)$$

where ε_0_ denotes the permittivity of the vacuum.

Recognizing the assumptions underlying model equations is crucial when applying them to real-world scenarios or specific systems. Often, we overlook these assumptions, which can lead to inaccurate interpretations or misapplications of the model. Therefore, careful consideration of the assumptions underlying these equations is essential for their proper use. The electromagnetic waves considered in these equations are applicable to stationary charges or stationary media [34]. The model assumes a uniform air gap across the layers. Additionally, it assumes uniform charge redistribution, constant temperature, ideal surface morphology, and no dielectric breakdown, which may not hold in real-world conditions.

### 2.3. Performance Factors

There are several factors that are essential for optimizing the performance of TENGs and enabling their widespread use in real-world applications. These factors—energy conversion efficiency, piezo/triboelectric coefficients, material innovation, and sustainable power sources—play a critical role in enhancing TENGs’ capabilities. High energy conversion efficiency ensures that TENGs generate sufficient power to operate small devices, which is crucial for wearable electronics, sensors, and IoT devices. Optimizing piezo/triboelectric coefficients through material innovation, such as using polymer gels, improves charge generation, allowing TENGs to efficiently harness energy from various mechanical stimuli. Furthermore, sustainable power sources like ambient energy harvesting enable TENGs to function autonomously, reducing the need for frequent recharging or external power supplies—an advantage for IoT and remote sensing applications [36]. By addressing these factors, TENGs can be designed to meet the increasing demand for self-powered, flexible, and sustainable devices across a wide range of industries.

#### 2.3.1. Energy Conversion Efficiency

Energy conversion efficiency is crucial for TENGs to effectively transform mechanical energy into electrical power. It is influenced by material properties, surface morphology, and the applied mechanical force. For TENGs to be effective in real-world applications [37,38], they must exhibit high efficiency to generate sufficient power for small devices. Materials with high triboelectric coefficients, such as polymer gels (hydrogels and ionogels), are preferred due to their flexibility and tunability, which enhance performance under deformation [17]. These materials also offer mechanical compliance [39], important for applications in wearable electronics and biomedical devices. To further enhance efficiency, strategies include optimizing the contact area between materials, adjusting surface roughness for improved electron affinity, and improving material conductivity. Additionally, integrating nanostructures or hybrid materials can boost the triboelectric effect, leading to higher energy output. Enhancing device design ensures maximum energy harvesting from various mechanical forces, increasing overall performance. Despite several efforts, the energy conversion efficiency of TENGs remains low. These losses are tied to the energy switches embedded in energy management circuits in TENGs. In a recent study [40], the energy conversion efficiency of TENGs has been increased by 8.5 times through a synergistic optimization technique between TENGs and switch configurations. This approach effectively mitigates voltage and charge losses caused by critical switches in the energy management circuits. Such strategies are vital for maximizing energy utilization efficiency in TENGs, especially given the ultra-high intrinsic impedance of these circuits. However, practical applications of this management efficiency have been limited, and the output regulation functionality is still underdeveloped. To tackle this, the study proposed a detailed energy transfer and extraction mechanism that mitigates voltage and charge losses. The efficiency was significantly improved by optimizing both the TENGs and switch configurations. Additionally, the study realized a TENG-based power supply with energy storage and regulation functions through advanced system circuit design, which enabled stable power delivery to electronic devices even under irregular mechanical stimuli. For instance, a rotating TENG that operates for only 21 s was able to power a hygrothermograph stably for 417 s. Furthermore, various types of TENGs were shown to consistently power devices like calculators and mini-game consoles, even when manually driven. This work advances the understanding of energy transfer and conversion in TENG systems and addresses the challenge of converting unstable mechanical energy into stable and usable electricity.

#### 2.3.2. Piezo/Triboelectric Coefficient

The piezoelectric and triboelectric effects are fundamental mechanisms in energy harvesting within TENGs [41]. The piezo/triboelectric coefficients signify a material’s ability to generate electrical charge under mechanical stress. The higher these coefficients, the more effectively the material converts mechanical energy into electrical energy, directly enhancing the TENG’s performance.

#### Material Selection

The piezoelectric properties of a material govern how effectively it converts mechanical deformation, such as pressure, stretching, or bending, into electrical charge. In contrast, the triboelectric properties are responsible for charge generation during the contact and separation of two materials with differing electron affinities. Materials like polytetrafluoroethylene (PTFE) [42], fluorinated ethylene propylene (FEP) [43], and silicone elastomers are commonly utilized in TENGs because they exhibit high piezoelectric and triboelectric coefficients, which contribute to their efficient energy harvesting capabilities.

#### Enhancing Coefficients

To improve the piezo/triboelectric coefficients, the incorporation of nanomaterials like carbon nanotubes (CNTs), graphene, and MXenes [23,44,45] is beneficial. These materials help to increase charge density and enhance the mechanical properties of TENGs, resulting in higher electrical output. Furthermore, hybrid materials that combine both piezoelectric and triboelectric effects can be designed to optimize energy conversion efficiency, providing improved versatility and performance.

#### Application Implications

Higher piezo/triboelectric coefficients contribute significantly to the scalability and efficiency of TENGs. Devices with improved coefficients can generate greater power output from minimal mechanical movements, which is particularly valuable for applications in wearable electronics, IoT sensors, and biomedical implants [46]. In these compact, power-limited environments, optimizing the piezo/triboelectric properties is crucial for ensuring reliable and continuous energy generation [47].

## 3. Materials-Based Strategies

Building upon these materials-based strategies, the subsequent sections of this review will delve into the detailed mechanisms behind each approach, exploring how they contribute to the overall performance optimization of TENGs. We will examine the latest advancements in surface morphological modifications and chemical treatments, highlighting their impact on charge density and energy conversion efficiency. Additionally, we will explore the role of material selection and structural design in preventing air breakdown, with a focus on innovative multi-layered architectures that enhance the stability and durability of TENGs. Finally, we will discuss the effective utilization of charge drift and its integration into TENG systems, aiming to maximize energy output and operational efficiency. These discussions will provide a comprehensive understanding of the key factors driving the development of high-performance TENGs for future applications in energy harvesting, wearable electronics, and IoT devices.

Before further exploring these strategies, it is crucial to first set the stage by understanding the fundamental principles of charge generation, which form the foundation for the subsequent materials and design improvements in TENG systems.

### 3.1. Fundamentals of Charge Generations in TENGs

TENGs convert mechanical energy into electrical energy through contact electrification [48] and electrostatic induction. Contact electrification occurs when two materials come into contact and separate, generating charge. This can occur even between identical materials with nonuniform surfaces. Understanding contact electrification, especially at solid–solid and solid–liquid interfaces, is key for optimizing TENG performances.

Though contact electrification has been studied for over 2600 years, the charge transfer mechanism is still unclear. Recent models focus on electron transfer, but they mainly apply to metal–semiconductor or metal–insulator interfaces [49], not metal–polymer or polymer–polymer systems. Additionally, ion transfer and material migration may also play a role in charge generation, particularly in polymer-based systems, suggesting a complex mix of physical and chemical factors.

To improve TENG output, increasing the transferred charge density is critical since both open-circuit voltage and short-circuit current depend on it. This density is influenced by the materials’ properties, such as their position in the triboelectric series. Efforts to enhance TENGs focus on selecting optimal materials and modifying their properties, including surface roughness, dielectric constants, and stretchability. Despite these advances, the maximum charge density achieved is about 260 μC/m^2^ for single-layer films and 283 μC/m^2^ for multilayer films [34,50].

To further understand how material properties influence charge transfer and density, it is essential to examine the underlying mechanisms of contact electrification, particularly in complex gel-based systems. The fundamental mechanisms of contact electrification in triboelectric nanogenerators remain an active topic of debate. Charge transfer during contact and separation may involve electron transfer, ion transfer, or material exchange, with the dominant mechanism largely dependent on the material system and interface conditions [34]. This complexity is particularly pronounced in gel-based systems, where the presence of liquids and mobile ions adds additional pathways beyond those observed in conventional solid polymers. In hydrogels, the high water content and ionic environment facilitate ion migration and electrochemical double-layer formation, contributing significantly to charge generation via ion transfer mechanisms. Ionogels, which incorporate ionic liquids as their swelling medium, predominantly exhibit ion–ion exchange and transport, further influencing the triboelectric response. In contrast, organogels swollen with organic solvents tend to behave more like traditional solid polymers, where electron transfer mechanisms prevail. Recent experimental and theoretical studies have started to elucidate these distinct charge transfer behaviors in gel-based materials [51,52]. However, a unified, comprehensive model describing contact electrification in gels remains elusive. Advancing mechanistic understanding in this area is crucial for rational design and optimization of gel-based TENGs with enhanced performance.

To resolve these outstanding mechanistic debates, we recommend the development of rigorous theoretical analyses combined with carefully designed experiments—such as controlled environmental studies, in situ spectroscopy, and interface-specific measurements—that can distinguish electron transfer from ion migration and material exchange in gel-based TENGs. A deeper grasp of these mechanisms will guide the development of novel gel materials with tailored interfacial properties, ultimately improving charge density and device efficiency.

**Figure 4:**
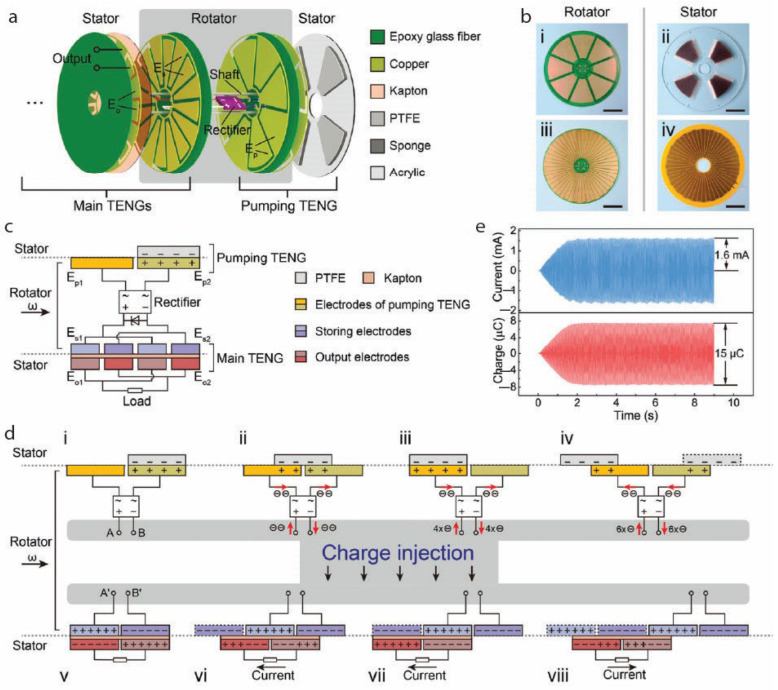
Schematic representation of charge pumping mechanism in TENGs. (**a**) Open view of the rotary charge pumping components and materials deployed in TENG. (**b**) Detailed photographs of the fabricated (**i**) rotator; (**ii**) the stator employed in a pumping TENG; (**iii**) the rotator; (**iv**) the stator of the main TENG; the scale bar used is 5 cm in each case. (**c**) Detailed visualizations of the circuit connections of the rotary-charge-pumping TENG. (**d**) (**i**–**iv**) The working principle of the charge-pumping TENG. (**v**–**viii**) The operating principle of the main TENG. (**e**) Short-circuit current and transferred charge of the device powered by one pair of pumping TENGs across four main TENG modes. Rotator with ω denotes the speed of rotation of the rotor in (**c**,**d**). Adapted with permission from ref. [53]. Copyright 2020, WILEY-VCH Verlag GmbH & Co. KGaA, Weinheim, Germany.

Building upon the understanding of charge transfer mechanisms, several advanced techniques have been developed to overcome inherent limitations in charge density and further enhance TENG output. To overcome this limitation and further boost charge transfer, advanced techniques such as charge pumping have been developed (Figure 4) [53]. Charge pumping involves using an auxiliary TENG, often designed as a rotary device, to actively shuttle charges to the main TENG, thereby enhancing its electrical output. Figure 4a illustrates the rotary charge TENG structure, combining a pumping TENG and a main TENG, each with rotator and stator disks hosting three electrode types: pumping electrodes (E_p_), charge storage electrodes (E_s_), and output electrodes (E_o_). Unlike conventional rotary TENG, the inversion design allows the pumping electrodes (E_p_) and storage electrodes (E_s_) to rotate synchronously, transferring charges efficiently via a rectifier without brushes or slip rings. The main TENG’s rotator and stator feature 48 complementary electrode sectors insulated by Kapton film adhered to the stator to prevent excessive surface polarization and output loss (Figure 4b,c). The pumping TENG generates alternating current converted to direct current by the rectifier, injecting opposite charges into E_s1_ and E_s2_ (Figure 4c), which induce corresponding charges in the output electrodes (E_o_), producing alternating current at the load (Figure 4d). This charge pumping mechanism decouples charge generation from friction intensity, allowing charge accumulation limited only by dielectric strength and stabilized by a Zener diode. As shown in Figure 4e, the rotary charge TENG achieves a short-circuit current of 1.6 mA and transferred charges of 15 µC at 2 Hz, significantly exceeding traditional rotary TENGs, thus enabling higher power output with reduced wear and enhanced device durability.

### 3.2. Materials Perspective

It is well known that material surfaces dominate electrochemistry, and TENGs are no exception. Surface properties such as roughness, dielectric constants, and stretchability significantly determine the efficiency of charge transfer and energy generation. Despite these advances, the maximum charge density achieved so far has not been at the commercial level. Therefore, it is essential to investigate and, at the same time, understand the means and methods to enhance it further.

#### 3.2.1. Gel-Based Polymer Composites

Gel-based polymer composites have emerged as a critical class of materials for TENGs due to their unique combination of mechanical flexibility, electrical performance, and biocompatibility. These gels typically consist of a three-dimensional (3D) polymeric network swollen with a liquid or ionic phase, which imparts remarkable softness and stretchability, allowing them to conform to complex, dynamic surfaces without loss of functionality. The primary types of gels used in TENG applications include hydrogels, ionogels, and organogels, each distinguished by their swelling medium and chemical composition.

Hydrogels are polymer networks swollen with water, offering high water content, excellent biocompatibility, and tunable mechanical properties. Their intrinsic softness and stretchability make them ideal candidates for wearable and implantable nanogenerators, particularly in biomedical sensing applications where conformal skin contact and comfort are paramount. Preparation methods for hydrogels often involve chemical or physical crosslinking of hydrophilic polymers, such as polyacrylamide or polyethylene glycol, which can be further enhanced by incorporating conductive fillers like carbon nanotubes (CNTs), graphene, or MXenes to improve electrical conductivity and charge transfer efficiency [2,54,55].

Ionogels replace water with ionic liquids as the swelling medium, combining the flexibility of gels with the intrinsic ionic conductivity and thermal stability of ionic liquids [56]. This makes ionogels especially promising for high-performance TENGs operating under harsh environmental conditions or wide temperature ranges. Ionogel preparation typically involves polymerizing monomers in the presence of ionic liquids or physically entrapping ionic liquids within a polymer matrix [57,58]. The high dielectric constant and stable electrochemical window of ionic liquids facilitate efficient charge separation and storage in TENG devices.

Organogels are swollen with organic solvents and offer distinct advantages in terms of chemical tunability and compatibility with hydrophobic triboelectric layers. They provide an alternative for applications where water-based hydrogels are unsuitable due to evaporation or freezing issues. Organogel synthesis generally employs polymerization or gelation of hydrophobic polymers in organic solvents, with possibilities to tailor mechanical and dielectric properties through polymer design and solvent selection [59].

Compared to conventional non-gel polymer materials such as polydimethylsiloxane (PDMS), polytetrafluoroethylene (PTFE), or fluorinated ethylene propylene (FEP), gel-based composites exhibit superior mechanical compliance, self-healing capability, and environmental adaptability. These properties enable gels to maintain high triboelectric output even under repetitive deformation, stretching, or bending—conditions where many traditional elastomers may fail or degrade. Moreover, the ability to embed conductive nanofillers within gels enhances their electrical properties, increasing charge density and energy conversion efficiency, as demonstrated in recent studies [2,3,60,61].

In summary, gel-based polymer composites represent a versatile and high-potential material platform for advancing the performance, durability, and application scope of triboelectric nanogenerators. Incorporating these materials into TENG design addresses many challenges related to mechanical flexibility, biocompatibility, and environmental stability, positioning gels as key enablers for next-generation wearable, implantable, and sustainable self-powered systems.

In a study, it was demonstrated that using a charge shuttling technique, charge density in TENGs can be significantly enhanced [62]. This novel TENG design differs from conventional models by utilizing the shuttling of charges within conduction domains rather than static charges confined to the dielectric surface. Driven by the interaction between two quasi-symmetrical domains, the shuttling of mirror charge carriers reportedly doubles the charge output. This mechanism results in an exceptionally high projected charge density of 1.85 mC/m^2^ under ambient conditions.

While this represents a substantial theoretical advancement, practical challenges—such as dielectric wear, material fatigue, and the difficulty of sustaining consistent charge shuttling over extended use—must be carefully considered. Moreover, the scalability and environmental robustness of such high-output configurations remain insufficiently explored.

**Figure 5:**
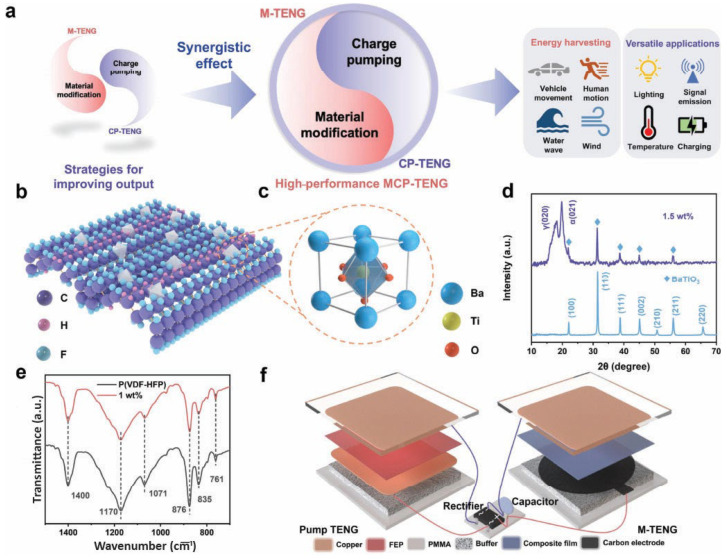
(**a**) Schematic representation of the fabrication process for high-performance TENGs. (**b**) Schematic showing P(VDF-HFP)/BTO composite film. (**c**) Chemical representation of BTO. (**d**) XRD of BTO and P(VDF-HFP)/BTO composite film. (**e**) The obtained FTIR spectra. (**f**) Replica of material-optimized charge-pumping TENGs. Adapted with permission from ref. [63]. Copyright 2024, Elsevier B.V.

Nevertheless, an integrated device for water wave energy harvesting demonstrates the practicality of this approach. The device offers valuable insights into new TENG modes, utilizing unfixed charges, and paves the way for high-performance mechanical energy-harvesting technologies. In the pursuit of higher output for large-scale industrialization of TENGs, an ultra-high-output TENG is proposed, inspired by synergistic effects found in nature [63]. This design combines internal material modification with external charge pumping integration (Figure 5a). The poly(vinylidene fluoride-co-hexafluoropropylene) (P(VDF-HFP)) film, with strong polarization and high permittivity, exhibits excellent triboelectric properties, which are further enhanced by doping with BaTiO_3_ (BTO) nanoparticles (Figure 5b,c). These structures were further verified by XRD pattern, as shown in Figure 5d. The addition of a charge pumping module allows for the efficient transfer of charges. As a result, a TENG made from an 8 µm P(VDF-HFP)/BTO 1 wt% composite film (verified by FTIR as shown in Figure 4e) achieves a remarkable total transfer charge density (TTCD) of 3.5 mC/m^2^ under optimal conditions. This was further fabricated in a device, as shown in Figure 5f, that surpasses that of both P(VDF-HFP)/BTO-based TENGs (0.1 mC/m^2^) and P(VDF-HFP)-based TENGs with charge pumping (2.6 mC/m^2^). This indicates the success of the proposed synergistic approach. Additionally, a novel charge transfer pattern based on this synergistic effect is introduced, highlighting the roles of material modifications and external charge pumping. This work presents an innovative concept for designing high-performance TENGs with ultra-high output. In another study [64], a triboelectric material charge transfer dynamic model is proposed, which enhances charge transfer efficiency. This dynamic charge transfer allows TENGs to achieve high surface charge density and improved device robustness, thereby boosting output power and extending device lifespan. The model not only deepens the understanding of high-performance TENGs but also provides valuable insights for optimizing and designing superior triboelectric materials. The triboelectric charge density is a key parameter for TENGs, but it is often reduced by charge escaping. A new study introduces a compensation method to suppress charge escaping in triboelectric polymers by inducing radical ion transfer [65]. Radical anions are injected into polytetrafluoroethylene (PTFE) to create ionized-PTFE (I-PTFE), which serves as the positive component for TENGs in combination with fully charged dielectrics. In open air, the TENGs achieved unprecedented charge densities of 525 μC/m^2^ in contact-separation mode and 1237 μC/m^2^ in sliding mode, both exceeding the air breakdown threshold. Secondary ion mass spectrometry confirms selective ion transfer at the contact interface, explaining the suppression of charge escaping. This strategy enables the maintenance of ultrahigh charge density in atmospheric conditions and provides insights into the complementary roles of electron and ion transfer in contact electrification.

Despite these material advancements, the long-term durability of TENGs remains a critical challenge. Repetitive contact-separation or sliding motions can lead to dielectric surface wear [66], mechanical fatigue, and gradual performance degradation. These issues are particularly significant for applications involving continuous mechanical deformation, such as wearable or implantable devices. While gel-based composites improve flexibility and stress tolerance, maintaining stable electrical output over extended cycles remains difficult. Strategies like self-healing gels, protective coatings, and encapsulation have shown promise, but comprehensive studies on fatigue resistance and wear under real-world conditions are still limited. Addressing these limitations is essential for the practical deployment and commercialization of high-performance TENG systems.

#### 3.2.2. Scalable Fabrication Strategies for Gel-Based TENGs

To realize commercial-scale applications of TENGs, scalable fabrication methods for gel-based materials are essential. Recent advances have shown promise in applying roll-to-roll processing, screen printing, and inkjet printing techniques to manufacture large-area, flexible TENG devices. For instance, Dhakar et al. [67] demonstrated a continuous roll-to-roll approach for patterning triboelectric layers with consistent performance across meters of material. Additionally, AI-assisted design frameworks are emerging to optimize material compositions and structural geometries. These systems leverage machine learning to predict output performance based on material inputs, significantly reducing trial and error in design cycles [68]. Together, these approaches enable high-throughput fabrication while maintaining precision and adaptability—key requirements for scalable, low-cost deployment of gel-based TENGs.

### 3.3. Phosphorus-Containing Organic–Inorganic Hybrid Materials

Organic–inorganic hybrid materials containing phosphorus have emerged as a promising class of compounds in energy storage research due to their unique combination of chemical versatility, structural stability, and electrochemical activity. These hybrids typically integrate phosphorus-based functional groups such as phosphates, phosphonates, or phosphazenes into organic frameworks or inorganic matrices, resulting in materials that leverage the advantages of both components. The phosphorus moieties often act as robust redox-active sites or structural stabilizers, enhancing ion transport and cycling stability in batteries and supercapacitors. In particular, phosphorus-containing hybrids demonstrate enhanced electrochemical performance owing to their strong chemical bonding, tunable electronic structures, and ability to form stable solid-electrolyte interphases. These features make them suitable for advanced lithium-ion and sodium-ion batteries, solid-state electrolytes, and multifunctional electrode materials [69]. Recent studies [70] have highlighted the synthesis of phosphorus-based organic–inorganic frameworks with improved capacity retention, rate capability, and thermal stability, positioning them as key candidates for next-generation energy storage devices.

Although direct integration with gel-based systems remains underexplored, the adaptable architectures and functional versatility of phosphorus-containing hybrids suggest strong compatibility with polymer matrices. Combining these hybrids with gel-based composites may enable the development of flexible, self-healing, and electrochemically active materials—attributes critical for wearable and soft energy devices.

Thus, while this review primarily focuses on polymer composites and nanogenerator materials, the inclusion of phosphorus-containing organic–inorganic hybrids broadens the discussion to highlight emerging hybrid material strategies with potential relevance to gel systems. Their integration into soft-matter frameworks represents a compelling direction for future multifunctional energy storage and conversion systems.

### 3.4. Other Materials

Nanomaterials such as carbon nanotubes (CNTs), graphene, and MXenes are widely used as conductive fillers in gel-based TENGs due to their exceptional electrical, mechanical, and surface properties [44,45,71]. Their inclusion in polymer gels significantly enhances charge transfer efficiency, dielectric behavior, and mechanical robustness, which are crucial for improving device performance and durability.

Carbon Nanotubes: CNTs possess a high aspect ratio, excellent electrical conductivity (~104–106 S/cm) [72], and remarkable tensile strength (~100 GPa), enabling them to form percolating conductive networks within gel matrices. This network facilitates rapid electron transport and enhances triboelectric charge density. Moreover, CNTs reinforce the mechanical structure of gels, improving tensile modulus and elongation at break, which allows TENGs to maintain stable output during repeated bending or stretching. For example, Lee et al. [73] developed a PDMS-based TENG incorporating ZnSnO_3_ (ZTO) nanostructures grown on surface-modified carbon nanotubes (SMCs). Their device with 0.3 wt% ZTO-SMC loading exhibited a remarkable output voltage of 665.63 V and current density of 137.08 mA/m^2^, representing improvements of 295% and 453% over pristine PDMS TENGs, respectively. The peak power density achieved was 10.57 W/m^2^ at a load resistance of 7 MΩ. Density functional theory calculations demonstrated that the enhanced TENG performance arises from the synergistic effects of increased dielectric constant, pressure-induced polarization, and an enlarged effective frictional area provided by the ZTO-SMC hybrid structure. This study provides critical insights into interface nanoengineering strategies for boosting the triboelectric performance of polymer-based TENGs, with promising implications for energy harvesting and self-powered systems.

Graphene: Graphene’s two-dimensional structure offers an exceptionally large surface area (~2630 m^2^/g), high intrinsic conductivity (~10^5^ S/m) [74], and excellent flexibility. These properties enable graphene to increase the dielectric constant of gels, enhance charge trapping, and improve electrical output. Graphene-based fillers also impart improved thermal stability and mechanical resilience to the gel, which is essential for maintaining consistent triboelectric performance under mechanical stress [75]. Hatta et al. [76] report the fabrication of a high-performance composite triboelectric nanogenerator (CTENG) utilizing a polydimethylsiloxane (PDMS) matrix doped with barium titanate (BTO) nanopowders and graphene quantum dots (GQDs) as conductive fillers. By varying the GQD content up to 40 wt%, they optimized the composite film, which was spin-coated on flexible ITO-coated polyethylene terephthalate (PET) substrates and thermally cured. The device with 30 wt% GQD demonstrated a significant enhancement in output performance, achieving an open-circuit voltage of ~310 V, a short-circuit current of ~23 μA, and a power density of 1.6 W/m^2^—approximately doubling the performance compared to composites without GQDs. The study highlights the synergistic effect of BTO’s dielectric properties and GQDs’ conductivity in boosting triboelectric output. The facile and scalable fabrication process indicates strong potential for PDMS/BTO/GQD composites in sustainable energy harvesting for small electronic devices.

MXenes: MXenes are a family of two-dimensional transition metal carbides and nitrides characterized by metallic conductivity (up to ~10^4^ S/cm) [77] and surface terminations (-OH, -O, -F) that enable strong interfacial interactions with polymer matrices. Their hydrophilicity and mechanical flexibility promote uniform dispersion and stable ion transport within gels. Wicklein et al. [78] investigated the impact of MXene chemical composition on triboelectric nanogenerator (TENG) performance by comparing Ti_3_CNT_x_ carbonitride with the more common Ti_3_C_2_T_x_ carbide in sodium alginate biopolymer composites. Adding 2 wt% Ti_3_CNT_x_ to alginate produced a synergistic enhancement in triboelectric output compared to Ti_3_C_2_T_x_. Spectroscopic and Kelvin probe force microscopy analyses revealed that the higher oxygen and fluorine surface content of Ti_3_CNT_x_ enhanced hydrogen bonding with alginate and increased surface charge density, resulting in a more negative surface potential that facilitated efficient charge transfer. The optimized Ti_3_CNT_x_-alginate TENG achieved outputs of 670 V, 15 μA, and 0.28 W/m^2^. Furthermore, plasma oxidation of MXene surfaces further improved performance. Importantly, Ti_3_CNT_x_-alginate exhibited tunable triboelectric polarity depending on the contacting material, acting as either tribopositive or tribonegative. This study advances understanding of MXene–biopolymer interactions and highlights the potential for tailored MXene-based TENGs with flexible, high-performance properties.

Future Directions:

Enhancing TENG performance hinges on advancing material surfaces, especially through gel-based polymer composites like hydrogels, ionogels, and organogels, which combine flexibility, biocompatibility, and high electrical output. Incorporating nanomaterials such as CNTs, graphene, and MXenes further boosts conductivity and durability. Emerging techniques like charge shuttling and charge pumping show promise in surpassing current charge density limits. Additionally, integrating phosphorus-containing organic–inorganic hybrids offers multifunctional improvements. Focused interdisciplinary research on material design, interface engineering, and scalable fabrication will be crucial to realizing durable, efficient, and commercially viable self-powered nanogenerators.

### 3.5. Preventing Air Breakdown

Air breakdown is a critical limitation in enhancing the output performance of TENGs, particularly as efforts are made to increase surface charge density. When the electric field between the triboelectric layers exceeds the dielectric strength of air, charge leakage occurs through ionization of air molecules, leading to significant energy loss and reduced device efficiency. To address this, various strategies have been developed [79,80]. These include engineering surface morphologies to distribute the electric field more uniformly, introducing insulating interlayers or solid dielectric spacers to suppress direct discharge paths, and operating TENGs under low-pressure or encapsulated environments to reduce ionization probability. Additionally, the use of high-permittivity materials or multilayered structures can lower the effective electric field per unit distance, thus mitigating breakdown risks. Such approaches are essential for maintaining high-voltage outputs and stable long-term operation, especially in high-performance or large-area TENG configurations. To achieve a high charge density with suppressed air breakdown, Liu et al. [79] demonstrated a significant enhancement in TENG performance by achieving an average charge density of 2.38 mC/m^2^ using a 4 μm PEI film and a carbon/silicone gel electrode under ambient conditions. Through both theoretical and experimental analysis, they revealed actual charge densities exceeding 4.0 mC/m^2^, highlighting the critical role of surface micro-contact efficiency and offering a promising strategy to further boost TENG output capabilities.

## 4. Applications

A TENG is a device that converts mechanical energy into electrical output, enabling the direct sensing of dynamic mechanical activities without the need for an external power input. This ability of TENGs allows them to be placed in the category of self-powered sensors. Recently, they have been utilized across a diverse range of applications, including electronic skin [81], including finger-touch detection [82], robotic arms [83], object motion tracking [84], MEMS displacement sensing [85], rotational sensors [86], and even chemical sensing [87].

### 4.1. TENGs as a Power Source

Triboelectric nanogenerators have rapidly evolved as promising sustainable energy solutions capable of powering various low-power electronic devices, particularly within sensor networks. This section highlights key examples demonstrating their practical implementation as efficient power sources.

#### 4.1.1. TENGs for Lighting and Power-Line Communication

**Figure 6:**
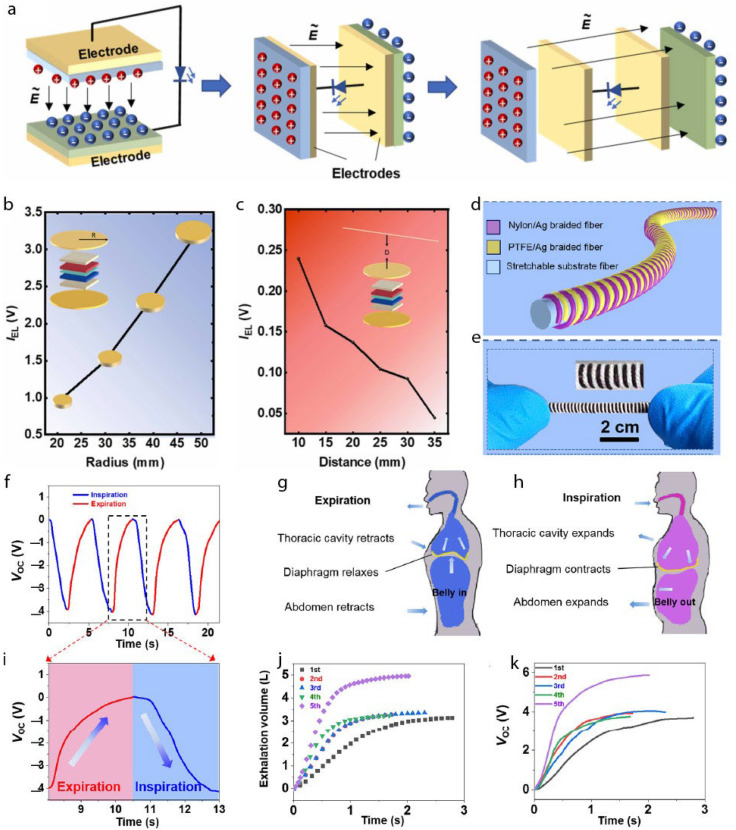
(**a**) Schematic representation of the device fabrication for TENG-driven LED for LIC operation. (**b**) Relationship showing the change in intensity over the electrode radius. (**c**) Data obtained for EL variations over working distance. Adapted with permission from ref. [91]. Copyright 2022, Elsevier. (**d**) Structural design of HFSS with a helical structure. (**e**) Photograph taken under a 0% strain condition. (**f**) Regular electrical signal as obtained during the procedure based on a chest trap of human breathing. (**g**,**h**) Schematic drawing showing the chest and abdomen during breathing, respectively. (**i**) Generated electrical signal of human breathing during complete respiratory cycle. (**j**) Spirometer measure of exhaled air. (**k**) The obtained V_oc_ plot over time. Adapted with permission from ref. [92]. Copyright 2022, American Chemical Society.

The primary goal of designing TENGs is to power small electronic devices engaged in sensor networks. TENGs as a power source were reported by Wang and co-workers in 2012 [88,89]. Since then, there has been a significant surge of interest in utilizing TENGs as sustainable energy sources for self-powered systems. A typical application of TENGs is powering LEDs [90]. Liao et al. [91] proposed an electrode–LED–electrode structure driven by TENG, namely, a LED-in-Capacitor (LIC) architecture designed to address the dual challenges of high-voltage monitoring and carrier signal demodulation in smart grid applications. Drawing inspiration from the operational principle of TENG-driven LEDs, the LIC employs a simple electrode–LED–electrode configuration capable of responding sensitively to changes in electric potential. The typical operation of TENG in CS mode is shown in Figure 6a. The fabricated device enables precise amplitude extraction and harmonic pollution detection on power lines, with a one-dimensional convolutional neural network achieving a classification accuracy of 94.53%. Figure 6b shows that the EL intensity increases with larger electrode radius due to enhanced electric field and charge accumulation. In Figure 6c, electroluminescence (EL) intensity decreases as the distance from the high-voltage (HV) line increases, indicating reduced electric field strength. Together, these results highlight the importance of electrode size and placement for optimizing LIC performance. Furthermore, the LIC facilitates accurate demodulation of high-frequency carrier signals, demonstrating its potential utility in power-line communication. As a novel fusion of TENG concepts and intelligent sensing, this work opens new pathways toward multifunctional, self-powered components for next-generation HV systems.

#### 4.1.2. TENGs for Wearable Respiratory Monitoring

Another essential yet crucial application of TENGs could be in human respiratory monitoring [84]. Ning et al. [92] fabricated a helical fiber strain sensor (HFSS) based on fiber-shaped triboelectric nanogenerators (FS-TENGs) to enable real-time, wearable respiratory monitoring (Figure 6d). Unlike traditional single-electrode FS-TENGs, which cannot sense their own strain, and coaxial double-electrode designs with limited sensitivity, the HFSS exhibits high responsiveness to subtle tensile strains due to its helical architecture. Figure 6e shows a photograph of HFSS under 0% strain. They further integrated the HFSS into a smart, self-powered respiratory monitoring system capable of detecting key breathing parameters for early disease diagnosis and triggering automated alerts via a preset mobile contact in response to abnormal respiratory patterns. As shown in Figure 6f, the HFSSs generate periodic electrical signals that correspond to the breathing cycle—expansion during inhalation and contraction during exhalation—mimicking diaphragm and abdominal movement (Figure 6g,h). To reduce environmental noise, the signals are filtered based on typical adult respiration rates (~16–20 breaths/min). The HFSS’s response to a full respiratory cycle is depicted in Figure 6i, while V_oc_ curves (Figure 6k) show strong alignment with exhalation volumes measured by a commercial spirometer (Figure 6j), confirming the system’s sensitivity and accuracy. Integrated into a self-powered wearable system, the HFSS can trigger automated alerts via a preset mobile contact upon detecting abnormal respiratory behavior. This work highlights the promise of FS-TENG-based helical structures for developing sensitive, self-powered respiratory monitoring devices for preventive healthcare.

### 4.2. TENGs as Sensors

Beyond their role as self-powered energy units, TENGs have also emerged as highly promising components in sensing applications. Their inherent ability to convert mechanical stimuli into electrical signals without external power makes them ideal candidates for a wide range of self-powered sensors. In recent years, TENG-based sensors have attracted considerable attention across various fields, including healthcare, environmental monitoring, human–machine interfaces, and structural health diagnostics. Among sensors, including optical, chemical, and temperature sensors, inertial measurement units (IMUs) and tactile sensors are particularly popular [36,93].

**Figure 7:**
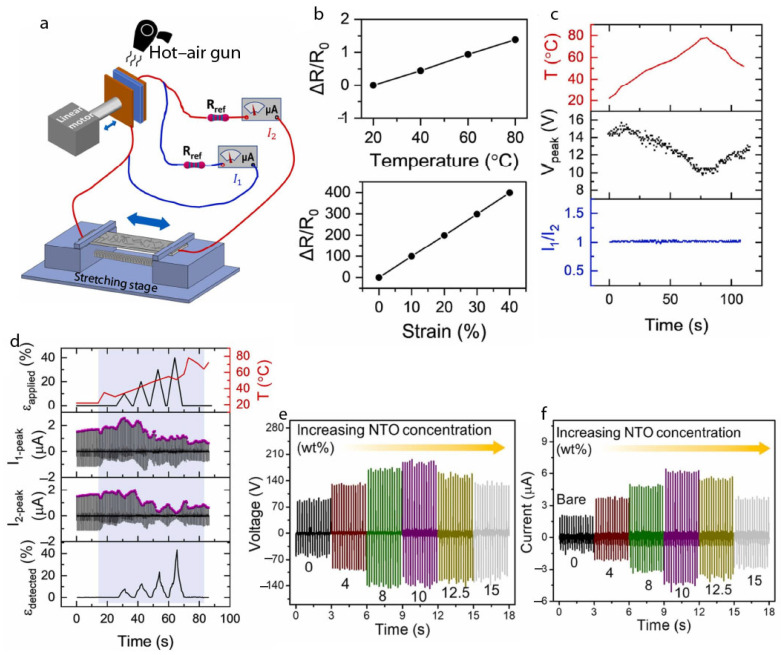
(**a**) Experimental setup of TENG-powdered strain sensor measurement under varying temperature. (**b**) Comparison of strain resistance change with respect to varying temperature (**top**) and varying strain (**bottom**). (**c**) Temperature variation in the TENG working environment was induced using a hot-air gun followed by natural cooling. The peak voltage and the current ratio between the two branches were measured under no-strain conditions. (**d**) Peak currents in the two branches and the corresponding calculated strain under varying applied strains at different temperatures. Adapted with permission from ref. [94]. Copyright 2023, Elsevier. (**e**) Voltage vs. time profile. (**f**) Current vs. time profile obtained for different TENGs consisting of sodium tantalate (NaTaO_3_ (NTO))/PDMS CFs loaded with varying amounts of NTO. Adapted with permission from ref. [95]. Copyright 2024, Elsevier.

Before moving to discuss the sensing applications of TENGs, it is essential to note that designed sensors must be insensitive to external variations; otherwise, the results would be unreliable. In this direction, Peng’s group [94] designed a novel self-powered sensing strategy to overcome the environmental variability issue inherent to TENGs. Recognizing that TENG output magnitude fluctuates with environmental changes, they proposed a current-ratio-based sensing method that splits the output into two branches—one containing a piezoresistive strain sensor and the other a reference resistor. The ratio of the currents, rather than absolute output, is used to extract the sensing signal, making the system insensitive to output fluctuations. This approach enables reliable, environment-invariant strain sensing, advancing the practical utility of TENG-powered IoT devices. The experimental setup looks like what is shown in Figure 7a. As mentioned earlier in this section, piezoelectric strain sensors are influenced by variation in temperature; the designed TENGs have a negligible variation as compared to strain, as can be seen in Figure 7b. As shown in Figure 7c, the peak output voltage decreases with rising temperature, though a slight initial increase near room temperature likely results from enhanced electron transfer due to a thermal gradient between the tribo-materials. As shown in Figure 7d, while the absolute current values vary with temperature, the current ratio—and thus the sensing performance—remains stable, accurately reflecting the applied strain. However, if currents drop too low at higher temperatures, measurement accuracy may suffer due to increased noise. This new method is anticipated to significantly improve the reliability and robustness of TENG-based self-powered sensing systems in practical applications. In this regard, Paranjape et al. [95] demonstrated a dual-function TENG system capable of simultaneous energy harvesting and sensing by integrating dielectric-modified polydimethylsiloxane (PDMS) films with voltage divider circuits. The optimized TENG exhibited stable electrical output (~195 V, ~6.27 µA) over 10,000 cycles, and resistive dividers were effectively used for real-time vehicle speed and direction detection, highlighting a simple and generalizable strategy for self-powered multifunctional sensing applications. The fabricated TENG with a bare PDMS film generated an electrical output of ~86 V, ~2 µA, and a charge density of ~32 µC/m^2^, serving as the baseline for further performance enhancements through filler incorporation (Figure 7e,f).

Expanding the scope of materials employed in TENG sensors, gel-based polymer composites such as hydrogels, ionogels, and organogels have shown great promise for enhancing sensor performance. Hydrogels, characterized by their high water content, flexibility, and biocompatibility, are especially suited for wearable and implantable TENG sensors that require conformal skin contact and comfort. For instance, Zhang et al. [96] reported an ionic hydrogel (PTSM) composed of polyacrylamide (PAM), tannic acid (TA), sodium alginate (SA), and MXene nanosheets, which exhibited ultra-high stretchability (>4600%), strong adhesion, and self-healing capabilities. The MXene enhanced the sensor’s electrical response, yielding a high gauge factor of 6.6. Silicone rubber-encapsulated hydrogel-based triboelectric nanogenerators (PTSM-TENGs) achieved an instantaneous power density of 54.24 mW/m^2^. A glove-based human–computer interaction system built with PTSM-TENGs enabled gesture visualization, robotic hand control, and object recognition with 98.7% accuracy using machine learning techniques, demonstrating significant potential for wearable sensing and intelligent control applications. Similarly, Huang et al. [97] developed a highly stretchable conductive hydrogel based on PEDOT:PSS, exhibiting excellent strain sensing performance with a gauge factor of 4.31, an ultra-wide sensing range (0–1690%), and a fast response time of 0.15 s. The hydrogel-based triboelectric nanogenerator (TENG) achieved an output voltage of up to 192 V. Integrated into wearable devices fixed on hands, wrists, legs, and feet, the TENG demonstrated effective human motion monitoring, highlighting its potential for strain sensors and self-powered wearable electronics.

Likewise, ionogels, containing ionic liquids within polymer networks, combine mechanical flexibility with intrinsic ionic conductivity and thermal stability, making them suitable for TENG sensors operating under challenging environmental conditions. Li et al. [98] used supramolecular chemistry and ZnO nanoparticle-reinforced poly(acrylic acid)-based self-healing ionogels to demonstrate high mechanical strength, compression resistance, transparency, and ionic conductivity under a wide operating temperature range. A self-healing ionogel-based triboelectric nanogenerator (SI-TENG) fabricated by sandwiching the ionogel between adhesive tape demonstrated a high output power density of 3.15 W/m^2^. The SI-TENG maintained reliable electrical performance across −30 to 80 °C and endured folding, twisting, stretching, and trampling. Importantly, the device restored full electrical output after mechanical damage through room-temperature self-healing, highlighting its potential for durable, flexible energy harvesting from human motion.

Similarly, organogels, swollen with organic solvents, offer chemical tunability and compatibility with hydrophobic triboelectric layers, providing stable sensor performance where water-based gels face limitations such as evaporation or freezing. Jing et al. [59] developed a novel triboelectric fiber (GS-fiber) incorporating an organogel conductor as a flexible, stretchable, and conductive electrode to overcome limitations of conventional electrodes in TENG textiles. The GS-fiber features a core/shell structure formed by photo-crosslinking the organogel within a transparent silicone hollow fiber mold, serving simultaneously as the gel electrode and the outer friction layer. This architecture addresses common issues such as cracking in metallic electrodes and leakage in liquid electrodes, enabling a stretchable and tailorable fiber suitable for wearable electronics. The facile fabrication process allows for scalable production, demonstrated by the preparation of a 30-meter-long GS fiber. When knitted into textiles (GS-TENG), these fibers efficiently harvest biomechanical energy, marking a significant advance toward the industrial application of TENG-based wearable textiles. Additionally, Zhao et al. [99] developed a dual-network conductive hydrogel composed of PEDOT:PSS, poly(vinyl alcohol), and carboxylated multi-walled carbon nanotubes, achieving a multifunctional triboelectric nanogenerator (DNH-TENG) with high stretchability (up to 566%) and excellent electrical performance, including an open-circuit voltage of 270.5 V and a short-circuit current of 16.2 μA. The dual-network design combines a conductive network and soft polymer matrix, enhanced by nanotube doping that improves conductivity and mechanical strength. The DNH-TENG sensor arrays demonstrated ultrahigh sensitivity for mechanical and temperature stimuli, enabling applications in pressure and temperature distribution monitoring.

Collectively, these gel-based materials enhance the mechanical compliance, electrical efficiency, and environmental robustness of TENG sensors, positioning them as key materials for next-generation self-powered sensing applications.

## 5. Problems to Be Discussed

As wearable electronics, flexible sensors, and self-sustainable systems continue to evolve, triboelectric nanogenerators (TENGs) have emerged as vital components in powering and sensing platforms [100,101]. Compared to conventional energy harvesters such as solar cells, thermoelectric generators, and piezoelectric nanogenerators, TENGs offer key advantages, including high power density, cost-effectiveness, flexibility, and ease of fabrication. Despite these merits, the practical deployment of TENGs still faces several significant challenges that must be addressed to unlock their full potential in real-world applications. In particular, gel-based polymer composites have shown promise as next-generation triboelectric materials, offering exceptional softness, flexibility, and tunable electrical properties. However, their integration within TENG systems presents unique challenges related to durability, environmental stability, and scalable manufacturing that require focused investigation.

### 5.1. Unclear Mechanisms of Contact Electrification

The underlying physics of contact electrification—central to TENG operation—remains incompletely understood. Competing theories, such as electron transfer, ion transfer, and material-dependent quantum effects, have been proposed, but no unified model exists. Polymer gels, with their tunable surface chemistry and unique interfacial dynamics, provide novel platforms to explore these mechanisms. The presence of ionic and liquid phases in gels influences charge transfer processes differently than conventional solid polymers, offering both opportunities and complexities in understanding electrification at the molecular level. Advancing this understanding is critical for designing gel-based TENG materials with predictable and optimized performance.

### 5.2. Material Durability and Surface Degradation

Triboelectric materials, especially polymers, are prone to mechanical wear, surface fatigue, and charge dissipation under repeated contact-separation cycles. This degradation limits long-term performance and scalability. Gel composites, including hydrogels and ionogels, exhibit intrinsic self-healing and excellent mechanical compliance that can mitigate these issues, improving resilience to mechanical damage. Nonetheless, maintaining stable electrical output under continuous mechanical stress and environmental exposure remains challenging. Approaches such as surface functionalization and the incorporation of nanofillers like graphene, carbon nanotubes (CNTs), and MXenes have demonstrated potential to enhance wear resistance and charge retention in gel matrices, yet require further optimization to realize reliable, durable TENGs.

### 5.3. Environmental Stability and Packaging

Environmental factors such as humidity, temperature, and pressure significantly affect TENG output, often causing inconsistent performance in wearable and outdoor scenarios. While hydrogels typically suffer from dehydration and swelling in varying humidity, ionogels and organogels offer superior environmental stability due to their ionic conductivity and hydrophobic nature. Advanced encapsulation techniques, such as superhydrophobic coatings and chemical crosslinking tailored for gel materials, are being developed to improve environmental robustness and ensure stable operation in diverse conditions.

### 5.4. Performance Enhancement Through Structure and Hybridization

Novel structural designs—such as grating, sponge-like, origami, and kirigami geometries—have shown promise in enhancing energy conversion and durability. Gel-based polymer composites enable the fabrication of stretchable, conformable, and self-healing architectures that maintain performance under large strains. Moreover, gels facilitate the integration of hybrid energy-harvesting approaches, combining triboelectric, piezoelectric, photovoltaic, and thermoelectric effects to deliver continuous, high-output power. These multifunctional gel-based platforms are pivotal in advancing flexible and wearable nanogenerator technologies.

To highlight the practical viability of TENGs in relation to other energy harvesting mechanisms, Table 1 provides a comparative overview of key performance characteristics across multiple technologies, including TENGs, electromagnetic generators (EMGs), piezoelectric nanogenerators (PENGs), solar cells, thermoelectric generators (TEGs), and pyroelectric nanogenerators (PyNGs). The comparison shows that TENGs typically achieve high open-circuit voltages and are uniquely suited for applications requiring high impedance compatibility, mechanical flexibility, and self-healing properties—particularly when gel-based materials are used.

As this review primarily focuses on TENGs, we include this chart specifically to benchmark TENG performance against PENGs, one of the most relevant mechanical energy-harvesting technologies. For a more detailed discussion on PENGs and other alternatives such as thermoelectric and photovoltaic harvesters, we kindly refer readers to the relevant literature [102,103,104].

**Table 1:** Comparison chart of different energy-harvesting technologies [104].

	Energy-Harvesting Technology					
	TENG	EMG	PENG	Solar Cell	TEG	PyNG
Working mechanism	triboelectrification and electrostatic induction	electromagnetic induction	piezoelectric effect	photovoltaic effect	Seebeck effect	pyroelectric effect
Open-circuit voltage	high	low	medium	low	low	medium
Short-circuit current	low	high	low	high	medium	low
Output power	low	high	low	high	medium	low
Power characteristic	AC	AC	AC	DC	DC	AC
Internal impedance	MΩ	Ω	Ω	Ω	Ω	kΩ

### 5.5. Scalability and Integration into Electronics

Integrating TENGs into large-area, flexible, and multifunctional electronics poses significant challenges, including signal conditioning, impedance matching, and energy storage integration. The soft and hydrated nature of gels adds complexity to fabrication and scaling efforts. However, emerging printing techniques, micro/nano-fabrication methods, and AI-assisted design optimized for gel materials show promise in overcoming these hurdles. Developing scalable, reproducible manufacturing processes for gel-based TENGs is crucial for transitioning from lab prototypes to commercial flexible electronics.

Overall, while gel-based materials present unique opportunities to enhance triboelectric nanogenerator performance and flexibility, addressing their durability, environmental resilience, and scalable integration remains essential to realize their full potential in practical applications.

### 5.6. Opportunities in Bioengineering and Health Monitoring

As the demand for self-powered biomedical systems continues to grow, TENGs are gaining increasing attention in the field of bioengineering, particularly for applications such as wearable health monitoring, implantable biosensors, and human–machine interfaces. These systems require energy autonomy, mechanical flexibility, and biocompatibility—traits well-aligned with the capabilities of TENGs. Among them, gel-based TENGs offer distinct advantages owing to their intrinsic softness, stretchability, and conformability, making them highly suitable for integration with soft biological tissues or dynamic skin surfaces.

Recent studies [105,106] have demonstrated that gel-based TENGs can effectively capture and convert biomechanical energy generated by human motion into electrical signals, enabling real-time monitoring of physiological activities such as heart rate, respiration, joint movement, and muscle contractions. These devices eliminate the need for bulky batteries, thus enhancing wearability, comfort, and long-term mobility. In implantable systems, biocompatible TENGs have been explored for applications like neural stimulation, cardiac pacing, and targeted drug delivery, where their self-powered nature can significantly reduce surgical complexity and improve patient safety.

Furthermore, the seamless integration of TENGs with flexible bioelectronic platforms, including stretchable sensors and wireless data transmission units, opens up new possibilities in personalized healthcare and prosthetic control. For instance, TENG-based sensors can enable prosthetic limbs to detect and respond to user intentions or surrounding stimuli, allowing for more intuitive control and feedback mechanisms.

These interdisciplinary innovations underscore the evolving role of TENGs—not merely as passive energy harvesters but as active, multifunctional components in intelligent biomedical systems. As research continues to advance, the convergence of materials science, bioengineering, and triboelectric energy harvesting will be critical in developing the next generation of self-powered, adaptive, and patient-centric medical devices.

## 6. Conclusions

This review presents a comprehensive analysis of TENGs, highlighting recent advances in materials design, charge generation mechanisms, and system integration for flexible, self-powered electronics. Strategies such as nanomaterial doping, surface engineering, and hybrid charge-pumping have shown significant potential in improving output performance. Critical challenges—including dielectric wear, environmental instability, charge escaping, and limited scalability—are addressed, with proposed solutions like surface functionalization, encapsulation techniques, roll-to-roll processing, and AI-assisted design offering promising directions. The review also emphasizes interdisciplinary opportunities, particularly in bioengineering and machine learning, where TENGs are being explored for wearable health monitoring, implantable sensors, and real-time adaptive systems. With sustained innovation in materials, modeling, and manufacturing, TENGs are well-positioned to become key components in next-generation biomedical and IoT platforms.
